# In Response to the Letter by Dramé et al.

**DOI:** 10.4269/ajtmh.18-0468b

**Published:** 2018-08

**Authors:** Frédérique Dorléans

**Affiliations:** E-mail: frederique.dorleans@santepubliquefrance.fr

Dear Sir,

We would like to address the two points raised in the letter related to our article “Outbreak of chikungunya in the French Caribbean islands of Martinique and Guadeloupe: findings from a hospital-based surveillance system (2013–2015).” The delay between the implementation of our hospital-based surveillance system in December 2013 and the production of the World Health Organization consensus case definitions in May 2015, referred to in this letter, explains why we could not use the new definitions. The methodology and tools used should be appreciated in light of this information, as described in our article. In fact, we comply with the principle of using international consensus case definitions. Unlike the shortened title of our article mentioned in the letter, suggesting a study in the general population, this manuscript mainly describes chikungunya virus (CHIKV) cases in a hospital setting. For instance, we do not report a 4% death rate in the overall population, as erroneously stated in the letter, but instead a 4% death rate in our cohort of CHIKV hospitalized cases. To conclude, we take this opportunity to emphasize the CHIKV-related severe and lethal cases that can occur during the course of a CHIKV epidemic to raise awareness for an infection that was first believed to be only a mild disease.

